# Development of a Rapid-Response Fluorescent Probe for H_2_S: Mechanism Elucidation and Biological Applications

**DOI:** 10.3390/bios15030174

**Published:** 2025-03-07

**Authors:** Trevor Dvorak, Haley Hernandez-Sandoval, Sunayn Cheku, Marijose Mora Valencia González, Linus Borer, Riley Grieser, Kimberly A. Carlson, Haishi Cao

**Affiliations:** 1 Department of Chemistry, University of Nebraska at Kearney, 2504 9th Ave, Kearney, NE 68849, USA; 2 Department of Biology, University of Nebraska at Kearney, 2504 9th Ave, Kearney, NE 68849, USAcarlsonka1@unk.edu (K.A.C.); 3 Facultad de Medicina Región Veracruz, Universidad Veracruzana, C. Agustín de Iturbide S/N, Zona Centro, Veracruz 91700, Mexico

**Keywords:** 1,8-naphthalimide, H_2_S, probes

## Abstract

Hydrogen sulfide (H_2_S) is an important signaling molecule involved in various physiological and pathological processes, making its accurate detection in biological systems highly desirable. In this study, two fluorescent probes (**M1** and **M2**) based on 1,8-naphthalimide were developed for H_2_S detection via a nucleophilic aromatic substitution. **M1** demonstrated high sensitivity and selectivity for H_2_S in aqueous media, with a detection limit of 0.64 µM and a strong linear fluorescence response in the range of 0–22 µM of NaHS. The reaction kinetics revealed a rapid response, with a reaction rate constant of 7.56 × 10^2^ M^−1^ s^−1^, and **M1** was most effective in the pH range of 6–10. Mechanism studies using ^1^H NMR titration confirmed the formation of 4-hydroxyphenyl-1,8-naphthalimide as the product of H_2_S-triggered nucleophilic substitution. **M1** was applied in MDA-MB-231 cells for cell imaging, in which **M1** provided significant fluorescence enhancement upon NaHS treatment, confirming its applicability for detecting H_2_S in biological environments. In comparison, **M2**, designed with extended conjugation for red-shifted emission, exhibited weaker sensitivity due to the reduced stability of its naphtholate product and lower solubility. These results demonstrate that **M1** is a highly effective and selective fluorescent probe for detecting H_2_S, providing a valuable resource for investigating the biological roles of H_2_S in health and disease.

## 1. Introduction

In biological systems, hydrogen sulfide (H_2_S) is an endogenous gas widely existing in various organs and tissues, including the cardiovascular system, gastrointestinal tract, immune cells, liver, and kidneys [1,2,3,4,5]. Endogenous H_2_S is produced via enzymatic pathways, in which cystathionine-β-synthase (CBS, EC 4.2.1.22), cystathionine-γ-lyase (CSE, EC 4.4.1.1), and 3-mecaptopyruvate sulfurtransferase (MST, EC 2.8.1.2) are the major contributors that produce endogenous H_2_S [6]. The concentration of H_2_S in human plasma is 10–300 μM, but it will vary in different locations [7]. As a signaling molecule, H_2_S is illustrated as a gasotransmitter and has gained significant attention alongside nitric oxide (NO) and carbon monoxide (CO) [8]. H_2_S exerts numerous physiological functions for immune response, signal transduction, and energy production [9,10,11]. However, paradoxical findings suggest that H_2_S exhibits both protective effects on cells and contributes to cell dysfunction and apoptosis [12,13,14]. To explain these contradictory facts, the cellular level of H_2_S is the most critical factor, which will determine the biofunctions of H_2_S, whether beneficial or deleterious [15]. Moreover, recent research indicated that the level of H_2_S is associated with major human diseases, such as Alzheimer’s disease, diabetes, and cancer [16,17,18]. Therefore, accurately measuring the level of H**_2_**S in living cells is the key factor in illustrating the pathophysiologic roles of H_2_S [19].

In the past decade, fluorescent probes have been intensively investigated as efficient approaches to detect H_2_S due to their high sensitivity, real-time response, low cost, and user-friendly operation [20,21,22]. Since H_2_S can dissociate into hydrosulfide (HS^−^) in aqueous media with a high dissociation rate (pK_a1_ = 7.0), many fluorescent probes have been developed based on the reaction with HS^−^, which shows a high nucleophilic character and reducing ability [23]. The most common strategies to design fluorescent probes for H_2_S detection include H_2_S-mediated azide and nitro group reduction, metal displacement, and nucleophilic aromatic substitution reactions [24,25,26,27]. Among these, the H_2_S-mediated azide reduction usually exhibited a relatively low rate because two equivalence HS^−^ are required to reduce the azide, forming a polysulfide intermediate [28]. Copper (Cu^2+^) is the most commonly used ion in the design of H_2_S probes based on metal displacement. Upon coordination with a metal chelator, Cu^2+^ induces fluorescence quenching. Adding HS⁻ displaces Cu^2+^ from the chelator, leading to precipitate formation and restoring the chelator’s fluorescence [29]. The sulfonyl esters and ethers based on 2,4-dinitrophenyl (DNP) and nitrobenzofurzan (NBD) platforms can efficiently react with HS^−^ via nucleophilic aromatic substitution reactions, which provides an effective mechanism to recognize H_2_S in different media [30,31]. After reacting with H_2_S, these reaction-based fluorescent probes generate a “Turn-On” or “Turn-Off” fluorescence response used for quantitative measurements of H_2_S [32,33].

Despite significant efforts to develop fluorescent probes for hydrogen sulfide (H_2_S) detection, numerous challenges remain. One major issue is the interference caused by reactive sulfur species (RSS), such as cysteine, glutathione, and polysulfides, during detection in biological environments [34]. Although RSS generally exhibit weaker nucleophilic properties and thus lower reactivity with reaction-based sensors, their high physiological concentrations (3–10 mM) can compensate for this limitation, leading to considerable interference [35]. Additionally, the inherent background fluorescence from biomolecules in biological samples further complicates detection [36]. A prolonged response time for reaction-based probes also limits their practical applicability [37].

To address these challenges, we report the development of novel fluorescent probes based on 1,8-naphthalimide (1,8-NI) for detecting H_2_S via nucleophilic aromatic substitution. These probes demonstrate high selectivity for H_2_S in aqueous media, even in the presence of biologically relevant species. Furthermore, cellular imaging studies confirm the excellent biocompatibility of these probes, enabling the effective measurement of cellular H_2_S in living organisms.

## 2. Experimental

### 2.1. General

All reagents used for synthesis and measurements were purchased in analytical grade from Sigma-Aldrich (St. Louis, MO, USA), Fisher Scientific (Pittsburgh, PA, USA), TCI (Portland, OR, USA), Alfa Aesar (Tewksbury, MA, USA), and Acros Organics (Waltham, MA, USA), and all were used as received unless otherwise specified. Absorption spectra were collected using a Cary Series UV–Vis Spectrophotometer (Agilent Technologies, Santa Clara, CA, USA). Fluorescence measurements were performed with a FluoroMax-4 Spectrofluorometer (Horiba Jobin Yvon, Irvine, CA, USA) using a 1 cm quartz cuvette. The excitation and emission slits were set between 1 and 5 nm. ^1^H and ^13^C NMR spectra were recorded on a Bruker 400 Ascend spectrometer at room temperature. All intermediates and final products were purified using a CombiFlash NextGen 300+ system (Teledyne ISCO, Lincoln, NE, USA). High-resolution mass spectrometry (HRMS) data were acquired at the Nebraska Center for Mass Spectrometry, University of Nebraska-Lincoln, using a GCT Mass Spectrometer (Waters, Milford, MA, USA). NaHS was used as the H_2_S source for solution-based measurements in this project. MDA-MB-231 epithelial cells (catalog #HTB-26) were obtained from ATCC (Manassas, VA, USA) for cell imaging experiments.

### 2.2. Synthetic Route and Characterization

**Scheme 1:**
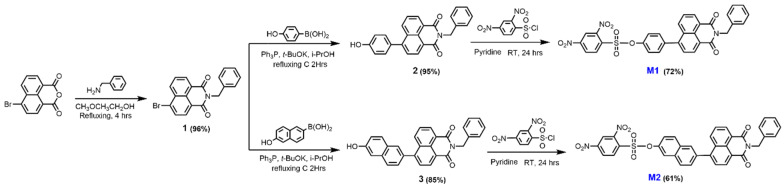
The synthetic routes to prepare **M1** and **M2**.

Compound **M1** and **M2** were synthesized via multiple-step reactions as shown in Scheme 1. ^1^H NMR and ^13^C NMR spectroscopy were used for characterization.

2-benzyl-6-bromo-1H-benzo[de]isoquinoline-1,3(2H)-dione (**1**): 4-Bromo-1,8-naphthalimide (138.0 mg, 0.5 mmol) was combined with phenylmethanamine (72.0 mg, 0.67 mmol) in ethanol (4.0 mL) and refluxed for 3 h. After completion of the reaction, the solvent was removed using a rotary evaporator. The resulting solid was purified by column chromatography using a mixture of dichloromethane and hexane (3:1). A white solid product (176 mg) was obtained as the product (96%). ^1^H NMR (400 MHz, DMSO-*d*_6_) δ: 5.25 (s, 2H), 7.21–7.41 (m, 5H), 8.01 (t, *J* = 8.0 Hz, 1H), 8.24 (d, *J* = 7.8 Hz, 1H), 8.37 (d, *J* = 8.0 Hz, 1H), 8.54–8.64 (m, 2H). ^13^C NMR (100 MHz, DMSO-*d*_6_) δ: 43.6, 122.4, 123.2, 127.6, 128.1, 128.9, 129.4, 129.9, 130.4, 131.8, 131.9, 132.4, 133.4, 137.6, 163.4, 163.5.

2-benzyl-6-(4-hydroxyphenyl)-1H-benzo[de]isoquinoline-1,3(2H)-dione (**2**): Compound **1** (183 mg, 0.5 mmol) was mixed with 4-hydroxyphenylboronic acid (89.7 mg, 0.65 mmol), triphenylphosphine (28.9 mg, 0.11 mmol), and t-BuOK (61.5 mg, 0.55 mmol) in isopropanol (5 mL). The mixture was heated at 80 °C for 2 h. Upon completion of the reaction, the mixture was poured into 12% HCl (50 mL) and extracted with dichloromethane (50 mL). The resulting yellow solid was further purified by column chromatography using a mixture of dichloromethane and ethyl acetate (9:1). The final product was obtained as a yellow powder, yielding 180 mg (95%). ^1^H NMR (400 MHz, DMSO-*d*_6_) δ: 5.28 (s, 2H), 6.99 (d, *J* = 8.2 Hz, 2H), 7.20–7.44 (m, 7H), 7.76 (d, *J* = 7.6 Hz, 1H), 7.86 (t, *J* = 7.6Hz, 1H), 8.35 (d, *J* = 8.2Hz, 1H), 8.51–8.58 (m, 2H), 9.88 (s, 1H). ^13^C NMR (100 MHz, DMSO-*d*_6_) δ: 43.3, 116.1, 120.8, 122.7, 127.5, 127.8, 127.9, 128.2, 128.6, 128.8, 129.1, 129.9, 131.2, 131.4, 131.7, 133.2, 137.8, 147.1, 158.4, 163.8, 164.0.

4-(2-benzyl-1,3-dioxo-2,3-dihydro-1H-benzo[de]isoquinolin-6-yl)phenyl 2,4-dinitrobenzene-Sulfonate (**M1**): Compound **2** (185 mg, 0.5 mmol) was dissolved in pyridine (2 mL) and reacted with 2,4-dinitrobenzenesulfonyl chloride (266 mg, 1 mmol) at 120 °C for 3 h. After completion of the reaction, the mixture was poured into a 12% HCl solution (20 mL) to precipitate the crude product. The crude product was purified by column chromatography using a mixture of dichloromethane and hexane (6:1). The final product (219 mg) was obtained as a white solid (72%). ^1^H NMR (400 MHz, DMSO-*d*_6_) δ: 5.27 (s, 2H), 7.08–7.47 (m, 7H), 7.61–7.70 (m, 2H), 7.81 (d, *J* = 7.4 Hz, 1H), 7.87 (t, *J* = 8.1 Hz. 1H), 8.18 (d, *J* = 8.6, 1H), 8.41 (d, *J* = 8.6 Hz, 1H), 8.50–8.61 (m, 2H), 8.67 (d, *J* = 8.0 Hz, 1H), 9.16 (s, 1H). ^13^C NMR (100 MHz, DMSO-*d*_6_) δ: 42.9, 121.5, 122.1, 122.8, 122.9, 127.5, 127.9, 128.0, 128.3, 128.4, 128.7, 128.8, 129.7, 131.0, 131.3, 131.6, 132.4, 132.5, 134.1, 137.7, 138.6, 144.9, 148.6, 149.0, 152.0, 163.7, 163.9.

2-benzyl-6-(6-hydroxynaphthalen-2-yl)-1H-benzo[de]isoquinoline-1,3(2H)-dione (**3**): Compound **1** (505 mg, 1.4 mmol) was mixed with (6-hydroxynaphthalen-2-yl)boronic acid (200 mg, 1.1 mmol), triphenylphosphine (58 mg, 0.22 mmol), and t-BuOK (123 mg, 1.1 mmol) in isopropanol (6 mL). The mixture was heated at 60 °C for 3 h. Upon completion of the reaction, the mixture was poured into 12% HCl (50 mL) to collect a yellow solid as the crude product. The yellow solid was further purified by column chromatography using pure dichloromethane. The final product was obtained as a yellow powder, yielding 503 mg (85%). ^1^H NMR (400 MHz, DMSO-*d*_6_) δ: 5.21 (s, 2H), 7.15–7.42 (m, 7H), 7.58 (d, *J* = 4.8 Hz, 1H), 7.83–7.95 (m, 4H), 8.01 (s, 1H), 8.37 (d, *J* = 4.8Hz, 1H), 8.54–8.63 (m, 2H), 9.99 (s, 1H). ^13^C NMR (100 MHz, DMSO-*d*_6_) δ: 43.4, 109.1, 127.0, 127.6, 127.9, 128.0, 128.1, 128.3, 128.6, 128.8, 128.9, 129.4, 130.0, 130.5, 131.2, 131.6, 132.9, 133.3, 134.8, 137.8, 147.2, 156.7, 163.9, 164.1.

6-(2-benzyl-1,3-dioxo-2,3-dihydro-1H-benzo[de]isoquinolin-6-yl)naphthalen-2-yl 2,4-dinitrobenzenesulfonate (**M2**): Compound **3** (140 mg, 0.33 mmol) was dissolved in pyridine (2.5 mL) and reacted with 2,4-dinitrobenzenesulfonyl chloride (217 mg, 0.82 mmol) at 110 °C for 3 h. After completion of the reaction, the mixture was poured into a 12% HCl solution (20 mL) to precipitate the crude product. The crude product was purified by column chromatography using a mixture of dichloromethane and ethyl acetate (3:1). The final product (132 mg) was obtained as a white solid (61%). ^1^H NMR (400 MHz, CDCl_3_) δ: 5.32 (s, 2H), 7.21–7.44 (m, 6H), 7.60 (d, *J* = 4.4 Hz 2H), 7.80 (d, *J* = 4.4 Hz, 1H), 7.88–8.00 (m, 3H), 8.18 (d, *J* = 4.4 Hz, 1H), 8.23–8.31 (m, 2H), 8.36 (d, *J* = 3.6 Hz, 1H), 8.50 (d, *J* = 4.8He, 1H), 8.59–8.69 (m, 2H), 8.98 (d, *J* = 1.2 Hz, 1H). ^13^C NMR (100 MHz, CDCl_3_-*d*_6_) δ: 43.8, 117.3, 119.0, 120.6, 122.1, 122.3, 123.0, 127.2, 127.5, 128.0, 128.2, 128.5, 128.8, 128.9, 129.0, 129.1, 129.2, 130.2, 131.1, 131.4, 131.5, 131.6, 132.5, 133.7, 136.9, 137.3, 139.9, 141.8, 146.2, 152.0, 155.9, 164.1, 164.3.

### 2.3. Cell Culture and Imaging

MDA-MB-231 epithelial cells (ATCC, Manassas, VA, USA) were grown in DMEM media containing 5% FBS, 100 µg /mL of streptomycin, and 100 IU/mL of penicillin for all experiments. The cells were incubated in a humidified atmosphere containing 5% CO_2_ set at 37 °C. For imaging, 600,000 cells were seeded in each well of a Cellvis 6 well glass bottom plate (Fisher Scientific, Pittsburgh, PA, USA, catalog #NC0452316). Upon reaching 75% confluency, cells were treated with 0–300 µM of an aqueous NaHS solution and left to incubate for an hour at 37 °C. Post-incubation, the media were removed, and the cells were washed with cold PBS followed by fresh DMEM media, on which a 10 µM **M1** probe (dissolved in DMSO) was added. Following a 45 min incubation period at 37 °C, media were removed from the wells, and cells were washed with cold PBS twice. All cell imaging was performed with wells containing PBS instead of DMEM to minimize background fluorescence.

All imaging was performed using an Olympus FV3000 laser scanning confocal microscope (Olympus, Tokyo, Japan). Images were taken using either a 20× or 60× oil immersion objective. Images for each sample were taken using the same microscope settings using a 405 nm laser at 0.2% transmissivity.

## 3. Result and Discussion

### 3.1. Synthesis

To synthesize probes **M1** and **M2**, commercially available 4-bromo-1,8-naphthalic anhydride first reacted with benzylamine to yield compound 1. Subsequently, Suzuki coupling reactions introduced phenyl and naphthalene groups at position 4 of compound **1**, forming compounds **2** and **3**. The coupling reaction yielded 95% and 85% yields for compounds **2** and **3**, respectively. Next, compounds **2** and **3** reacted with 2,4-dinitrosulfonyl chloride to yield the desired probes **M1** and **M2**.

### 3.2. Photophysical Properties of **M1** and **M2**

The photophysical properties of compounds **M1** and **M2** were investigated in five different solvents: DMSO, MeCN, acetone, MeOH, and THF, as summarized in Table 1. Both compounds exhibited significant solvatochromic behavior, with absorption maxima observed in the 345–363 nm range and fluorescence emission maxima in the 422–501 nm range.

The dinitrobenzene sulfonate group in **M1** and **M2** exhibited a markable fluorescence quenching effect, resulting in low quantum yields, particularly in polar solvents such as DMSO. Notably, **M2**, which features an extended naphthalene moiety with increased π-conjugation compared to **M1**, displayed bathochromic shifts in both absorption and emission spectra. The increased conjugation significantly contributed to the red shift, highlighting the critical influence of structural modifications on their optical characteristics.

### 3.3. Quantitative Detection of H_2_S

**Figure 1:**
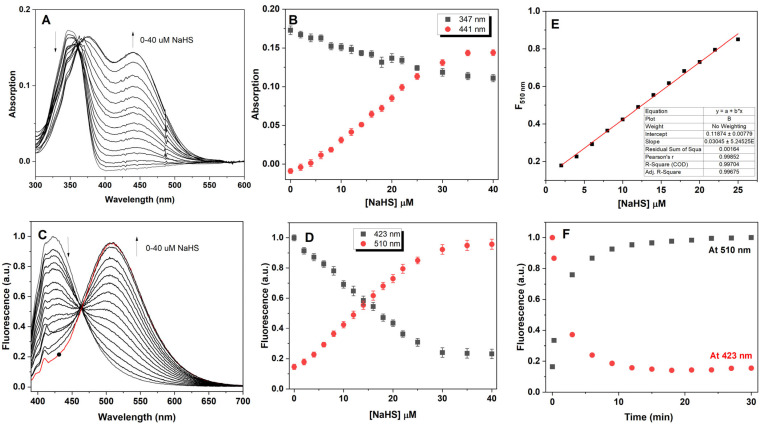
**M1** (10 μM) is titrated with NaHS (0–40 μM) in a 1:1 (*v*/*v*) mixture of MeCN/H_2_O at room temperature. Spectra are recorded after 20 min of incubation. (**A**) Absorption spectra of **M1** in the presence of increasing concentrations of NaHS. (**B**) Changes in the absorption of **M1** at 347 nm and 441 nm. (**C**) Fluorescence spectra of **M1** with increasing concentrations of NaHS (λ_ex_ = 365 nm). (**D**) Changes in fluorescence emission of **M1** at 423 nm and 510 nm. (**E**) Linear correlation between fluorescence intensity at 510 nm and NaHS concentration. (**F**) After incubating **M1** (10 μM) with NaHS (40 μM) in a 1:1 (*v*/*v*) mixture of MeCN/H_2_O at room temperature, the fluorescence emission changes at 423 nm and 510 nm stabilize within 10 min.

To assess the sensing capability of **M1** to H_2_S, **M1** (10 μM) was incubated with different amounts of NaHS (0–40 μM) for 20 min at room temperature. Due to the high dissociation rate of H_2_S in aqueous media, NaHS was utilized as the H_2_S resource for all measurements. The absorption and fluorescence emission spectra were collected after 20 min incubation, as shown in Figure 1. To consider balancing signal strength and biocompatibility, a 1:1 (*v*/*v*) mixture of acetonitrile and water (MeCN/H_2_O) was chosen as the medium to conduct these measurements.

The maximum absorption of free **M1** was observed at 347 nm. As a sulfonate ester, **M1** showed a high reaction rate with NaHS via a nucleophilic aromatic substitution reaction to yield a phenol derivative, leading to a significant change in both absorption and emission spectra. In the presence of NaHS, the maximum absorption changed from 347 nm to 376 nm, indicating the formation of 4-hydroxyphenyl-1,8-naphthalimide. Notably, a new absorption peak at 441 nm was observed, corresponding to the phenolate species generated by the rapid deprotonation of 4-hydroxyphenyl-1,8-naphthalimide (Figure 1A,B).

The maximum fluorescence emission spectra were observed at 423 nm with an excitation at 365 nm. Following the addition of NaHS, the emission peak of **M1** at 423 nm rapidly decreased, while a new emission peak appeared at 510 nm. This spectral shift corresponds to the formation of 4-hydroxyphenyl-1,8-naphthalimide via the H_2_S-triggered substitution reaction (Figure 1C,D). Interestingly, no fluorescence emission was observed when the absorption wavelength at 441 nm was used as the excitation.

Absorption and emission spectral analysis confirmed the high sensitivity of **M1** to NaHS. Upon the addition of 40 μM of NaHS, spectral changes reached a plateau, indicating the completion of the H_2_S-triggered nucleophilic aromatic substitution reaction. Moreover, the fluorescence emission intensity at 510 nm exhibited a strong linear correlation with NaHS concentrations in the 0–22 μM range (Figure 1E). The limit of detection (LOD) was calculated to be 0.64 μM (0.0218 ppm) using the equation LOD = 3*δ*/*S*, where *δ* is the standard deviation and *S* is the slope of the calibration curve.

Fluorescence emission spectra were used to investigate the reaction kinetics of **M1** with NaHS under identical experimental conditions. Upon mixing NaHS (40 μM) with **M1** (10 μM) in a 1:1 (*v*/*v*) MeCN/H_2_O solution at room temperature, fluorescence spectra were recorded over 30 min (λ_ex_ = 365 nm). After initiating the reaction, the emission at 432 nm decreased rapidly, while a concurrent emission enhancement at 510 nm was observed. Both intensity changes reached a plateau within 10 min, indicating reaction completion (Figure 1F). Kinetic analysis using a pseudo-second-order model yielded a reaction rate constant of 7.56 × 10^2^ M^−1^ s^−1^. Compared to other reaction-based H_2_S probes, **M1** showed a short response time to H_2_S (Table S1).

### 3.4. Selectivity of **M1** to H_2_S

**Figure 2:**
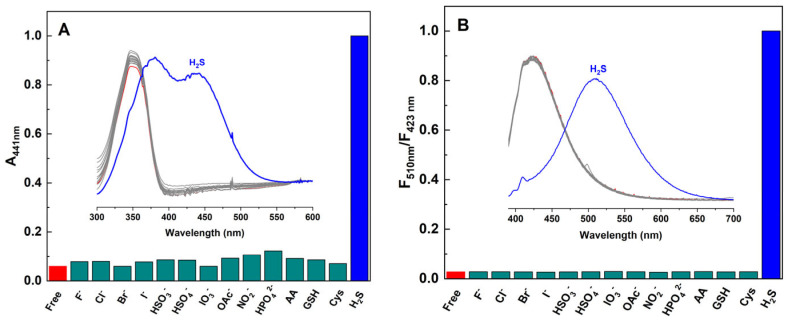
**M1** (10 μM) is incubated with a series of potential interferents, including F^−^, Cl^−^, Br^−^, I^−^, HSO_3_^−^, HSO_4_^−^, IO_3_^−^, OAc^−^, NO_2_^−^, HPO_4_^−^, ascorbic acid, GSH, and Cys (40 μM) to evaluate its selectivity for H_2_S. The reactions are conducted in a 1:1 (*v*/*v*) mixture of MeCN/H_2_O at room temperature, and spectra are recorded after 20 min of incubation. (**A**) Absorption spectra of **M1** in the presence of various species. (**B**) Fluorescence spectra of **M1** in the presence of various species (λ_ex_ = 365 nm). Blue lines represent the spectra of **M1** in the presence of H_2_S, while the gray lines correspond to the spectra of free **M1** and its interactions with F^−^, Cl^−^, Br^−^, I^−^, HSO_3_^−^, HSO_4_^−^, IO_3_^−^, OAc^−^, NO_2_^−^, HPO_4_^−^, ascorbic acid, GSH, and Cys.

To evaluate the selectivity of **M1** for H_2_S detection, 13 nucleophilic species (F^−^, Cl^−^, Br^−^, I^−^, HSO_3_^−^, HSO_4_^−^, IO_3_^−^, OAc^−^, NO_2_^−^, HPO_4_^−^, ascorbic acid, GSH, and Cys) were tested under identical conditions. Each species (40 μM) was incubated with **M1** (10 μM) in a 1:1 (*v*/*v*) MeCN/H_2_O mixture at room temperature for 20 min, and the absorption and emission spectra were recorded. As shown in Figure 2, only H_2_S induced a remarkable red shift in both the absorption and emission spectra, while all other species showed negligible spectral changes. This demonstrates that **M1** exhibits high selectivity toward H_2_S due to its specific nucleophilic aromatic substitution mechanism. The absence of interference from biologically relevant nucleophiles supports the probe’s potential for accurate H_2_S detection in complex systems.

### 3.5. pH Effect on the Sensing Capability of **M1**

**Figure 3:**
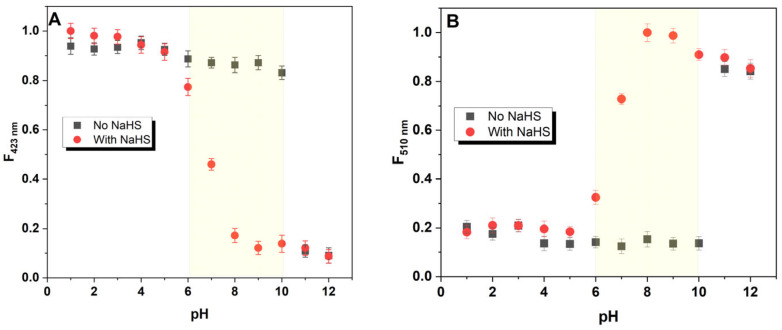
The pH-dependent H_2_S sensing of **M1** (10 μM) is evaluated in 1:1 MeCN/buffer (pH 1–12) with 40 μM H_2_S: (**A**) Fluorescence emission changes in **M1** at 423 nm are monitored in the presence and absence of H_2_S (λ_ex_ = 365 nm). (**B**) Fluorescence emission changes in **M1** at 510 nm are recorded with and without H_2_S (λ_ex_ = 365 nm).

As a sulfonate ester, **M1** is sensitive to changes in pH due to the potential hydrolysis of the ester group. Additionally, **M1** is designed for quantitatively detecting H_2_S, whose nucleophilicity is highly pH-dependent. This pH dependency significantly influences the reaction rate between **M1** and H_2_S, thereby affecting the probe’s response time. To investigate the pH-dependent sensing capability, **M1** (10 μM) was measured in a 1:1 (*v*/*v*) mixture of MeCN and pH buffer (pH 1–12) at room temperature. Fluorescence spectra were recorded after 20 min of incubation. Theoretically, **M1** is expected to exhibit fluorescence quenching at 423 nm and enhancement at 510 nm upon reacting with NaHS. The pH-dependent fluorescence response of **M1** in the presence of NaHS was investigated across the pH range of 1–12 (Figure 3). In media with pH 1–6, no significant emission changes were observed at 423 nm or 510 nm, likely due to the protonation of HS^−^, which reduced its nucleophilicity and slowed the reaction with **M1**. In contrast, at pH 6–10, the addition of NaHS to **M1** resulted in fluorescence quenching at 423 nm and enhancement at 510 nm, with the strongest response observed at pH 9. This pH condition provided the maximum fluorescence signal for H_2_S detection. At pH 10–12, fluorescence changes at 423 nm and 510 nm were detected even in the absence of NaHS, indicating that OH^−^ reacted with **M1**, causing similar spectral changes. These results suggested that pH 6–10 is the optimal pH range for **M1** to detect H_2_S with minimal interference.

### 3.6. The Sensing Mechanism for H_2_S

**Scheme 2:**
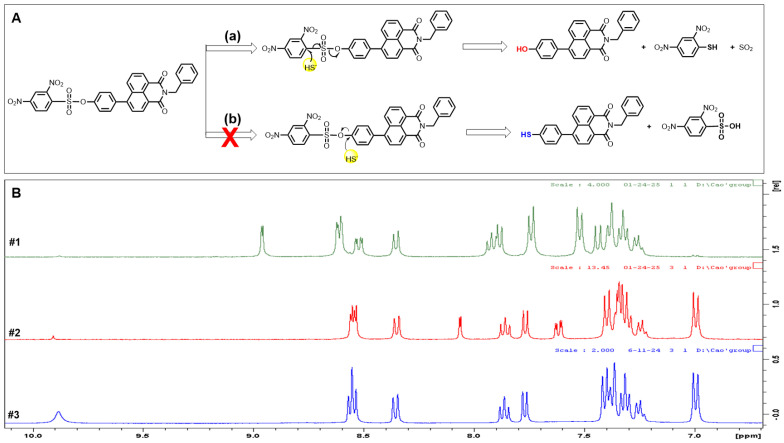
(**A**) Two proposed reaction mechanisms for the nucleophilic substitution employed by **M1** for detecting H_2_S. Theoretically, H_2_S can attack dinitrobenzene moiety (a) or naphthalene ring (b) to complete the substitution reaction. The ^1^H NMR titration data supported mechanism (a). (**B**) The ^1^H NMR titration for **M1** with the addition of NaHS in DMSO-*d*_6_/H_2_O.

Since **M1** employs nucleophilic substitution as its sensing mechanism for H_2_S detection, the nucleophile (H_2_S) can potentially target two reactive sites: the dinitrobenzene moiety or the phenyl ring attached to the 1,8-naphthalimide core, leading to distinct products and fluorescence emissions (Scheme 2). To confirm the H_2_S-triggered nucleophilic substitution mechanism of **M1**, ^1^H NMR titration experiments were performed. As shown in Scheme 2, the ^1^H NMR spectra were obtained for **M1** (Panel #1), a mixture of **M1** and NaHS (Panel #2), and 4-hydroxyphenyl-1,8-naphthalimide (Panel #3). Upon reacting with NaHS, **M1** yielded a product with an identical ^1^H NMR spectrum to 4-hydroxyphenyl-1,8-naphthalimide, including a characteristic peak at 9.90 ppm corresponding to the phenol group. These results conclusively demonstrate that **M1** undergoes a nucleophilic substitution reaction with NaHS, using mechanism (a) to yield 4-hydroxyphenyl-1,8-naphthalimide as the product.

### 3.7. Detection of Cellular H_2_S in MDA-MB-231 Cells

**Figure 4:**
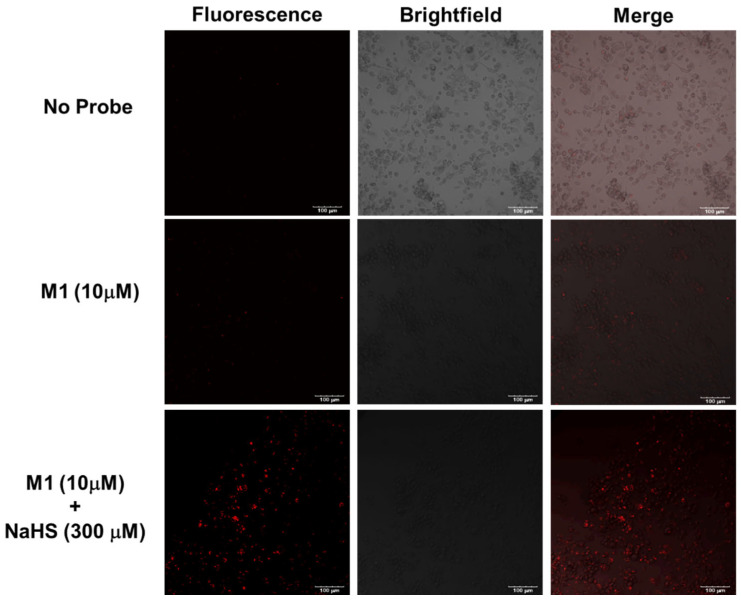
Apply **M1** (10 μM) in MDA-MB-231 cells for cell imaging. NaHS (300 µM) is incubated with **M1** for 45 min incubation at 37 °C. UV filter is used with an excitation at 365 nm.

MDA-MB-231 epithelial cells (ATCC, Manassas, VA, USA) were cultured in DMEM supplemented with 5% FBS, 100 μg/mL of streptomycin, and 100 IU/mL of penicillin and incubated at 37 °C with 5% CO_2_. For imaging experiments, 600,000 cells were seeded into each well of a Cellvis 6-well glass-bottom plate (Fisher Scientific, Pittsburgh, PA, USA; catalog #NC0452316) until they reached approximately 75% confluency. At this time, the cells were treated with a 0–300 μM aqueous NaHS solution for 1 h at 37 °C, and the media were aspirated and washed with cold PBS. After the wash, the cells were incubated with 10 μM of **M1** in DMEM for 45 min at 37 °C. Subsequently, the media were removed, cells were washed twice with cold PBS, and imaging was performed. To reduce background fluorescence, imaging was performed using wells containing PBS instead of DMEM. The results show that **M1** exhibited no detectable fluorescence in MDA-MB-231 cells, but a significant fluorescence enhancement was observed following the addition of 300 μM of NaHS (Figure 4), therefore demonstrating that **M1** functioned effectively as a probe for detecting H_2_S in a cellular environment.

### 3.8. Sensing Capability of **M2**

**Figure 5:**
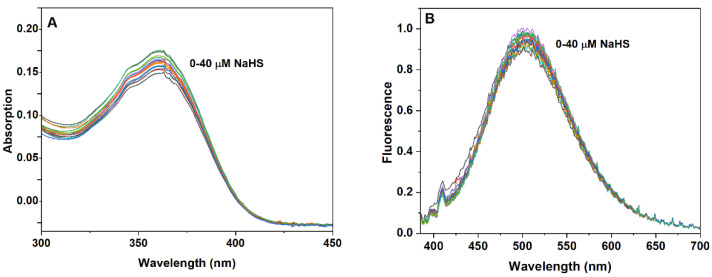
**M2** (10 μM) is incubated with NaHS (0–40 μM) in a 1:1 (*v*/*v*) mixture of MeCN/H_2_O at room temperature. Spectra are recorded after 20 min of incubation. (**A**) Absorption spectra of **M2** in the presence of increasing concentrations of NaHS. (**B**) Fluorescence spectra of **M2** in the presence of increasing concentrations of NaHS.

To compare with **M1**, **M2** was synthesized with extended conjugation, aiming to achieve fluorescence emission at a longer wavelength. The sensing performance of **M2** was evaluated under the same experimental conditions as **M1**. **M2** (10 μM) was titrated with NaHS (0–40 μM) at room temperature, and the absorption and emission spectra were collected after 20 min of incubation in a 1:1 MeCN/H_2_O mixture (Figure 5). **M2** displayed an absorption at 361 nm and an emission at 511 nm. However, neither absorption nor emission exhibited significant changes in response to NaHS within the tested concentration range (0–40 μM).

Compared to **M1**, **M2** showed significantly weaker sensitivity to H_2_S, likely because it reacts with H_2_S to yield a sulfonate ester rather than producing SO_2_ and naphthol. The presence of the sulfonyl group in the product induced a quenching effect, leading to a reduced fluorescence response. Additionally, **M2** showed lower solubility in MeCN compared to **M1**, which may further contribute to its low sensitivity to H_2_S. Due to its poor sensitivity to H_2_S, a pH-dependent sensing study was not conducted for **M2**.

## 4. Conclusions

In this study, we developed and characterized two novel 1,8-naphthalimide-based fluorescent probes, **M1** and **M2**, for the selective detection of H_2_S in biological systems. **M1** exhibited a high sensitivity and selectivity for H_2_S, with a detection limit of 0.64 µM and a rapid response rate, attributed to its efficient nucleophilic aromatic substitution mechanism. **M1** showed distinct spectral changes in absorption and emission spectra in the presence of H_2_S, making it a tool with multiple windows for the quantitative detection of H_2_S in aqueous media over a physiologically relevant pH range (pH 6–10). Mechanism studies were conducted using ^1^H NMR titration, confirming the formation of a phenol derivative rather than a thiol derivative as the reaction product. Moreover, **M1**’s biocompatibility and ability to detect H_2_S in living MDA-MB-231 cells further validated its potential as a practical tool for studying H_2_S in biological environments. In contrast, **M2**, designed with extended conjugation for red-shifted fluorescence, displayed lower sensitivity to H_2_S due to the reduced stability of its reaction product and decreased solubility in the aqueous medium. These findings provided a valuable tool for advancing our understanding of the physiological and pathological roles of H_2_S in major human diseases.

## Figures and Tables

**Table 1:** The photophysical properties of **M1** and **M2** in different solvents.

	M1				M2			
	λ_ab_	ε	λ_em_	Φ	λ_ab_	ε	λ_em_	Φ
	(nm)	(M^−1^ cm^−1^)	(nm)		(nm)	(M^−1^ cm^−1^)	(nm)	
**DMSO**	352	14,220	422	0.007	363	18,720	483	0.008
**MeCN**	345	19,440	423	0.027	361	23,220	478	0.010
**Acetone**	345	19,260	492	0.042	357	24,180	425	0.002
**MeOH**	348	21,180	431	0.028	359	11,820	436	0.037
**THF**	346	20,520	474	0.077	355	25,020	421	0.028

## Data Availability

Data are contained within the article.

## Supplementary Materials

The following supporting information can be downloaded at https://www.mdpi.com/article/10.3390/bios15030174/s1, Table S1: The comparison of detection parameters of **M1** with other H_2_S probes. References [38,39,40,41,42] are cited in the Supplementary Materials.
