# Supplementary material for: Development of a Rapid-Response Fluorescent Probe for H2S: Mechanism Elucidation and Biological Applications
Source: Biosensors (Basel). 2025 Mar 7;15(3):174. doi: 10.3390/bios15030174 (PMC11940642; doi:10.3390/bios15030174)
Supplement: Supplementary file 1 [file biosensors-15-00174-s001.zip › biosensors-3483939-supplementary.pdf]

Development of a Rapid-Response Fluorescent Probe for H_2S: Mechanism Elucidation and Biological Applications

Trevor Dvorak[1], Haley Hernandez-Sandoval[1], Sunayn Cheku[2], Marijose Mora Valencia Gonzalez [3], Linus Borer[1], Riley Grieser[1], Kimberly A. Carlson[2], Haishi Cao[1]*

1. Department of Chemistry, University of Nebraska at Kearney, 2504 9th Ave, Kearney, NE 68849, United States of America
2. Department of Biology, University of Nebraska at Kearney, 2504 9th Ave, Kearney, NE 68849, United States of America
3. Universidad Veracruzana, C. Agustín de Iturbide S/N, Zona Centro, 91700 Veracruz, Ver. Mexico

* Correspondence: caoh1@unk.edu

NMR spectra

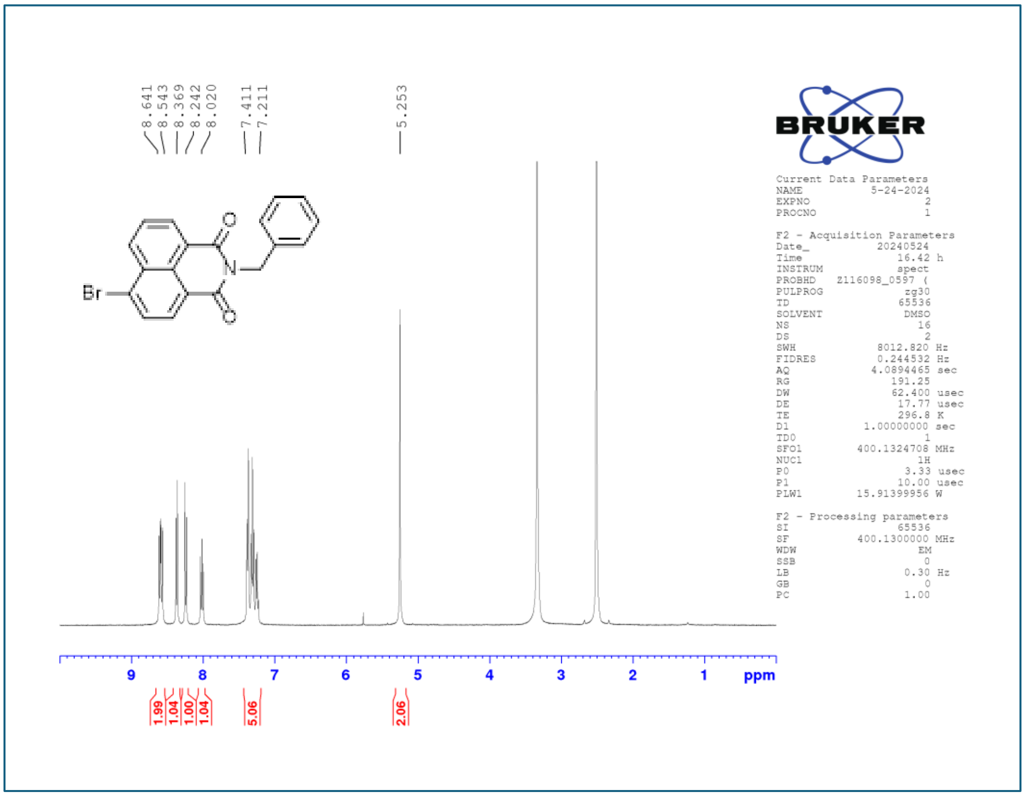
8.641
8.543
8.369
8.242
8.020
7.411
7.211
5.253
Br
O
N
BRUKER
Current Data Parameters
NAME 5-24-2024
EXPNO 2
PROCNO 1
F2 - Acquisition Parameters
Date_ 20240524
Time 16.42 h
INSTRUM spect
PROBHD Z116098_0597 (
PULPROG zg30
TD 65536
SOLVENT DMSO
NS 16
DS 2
SWH 8012.820 Hz
FIDRES 0.244532 Hz
AQ 4.0894465 sec
RG 191.25
DW 62.400 usec
DE 17.77 usec
TE 296.8 K
D1 1.00000000 sec
TD0 1
SFO1 400.1324708 MHz
NUC1 1H
P0 3.33 usec
P1 10.00 usec
PLW1 15.91399956 W
F2 - Processing parameters
SI 65536
SF 400.1300000 MHz
WDW EM
SSB 0
LB 0.30 Hz
GB 0
PC 1.00
9 8 7 6 5 4 3 2 1 ppm
1.99
1.04
1.00
1.04
5.06
2.06

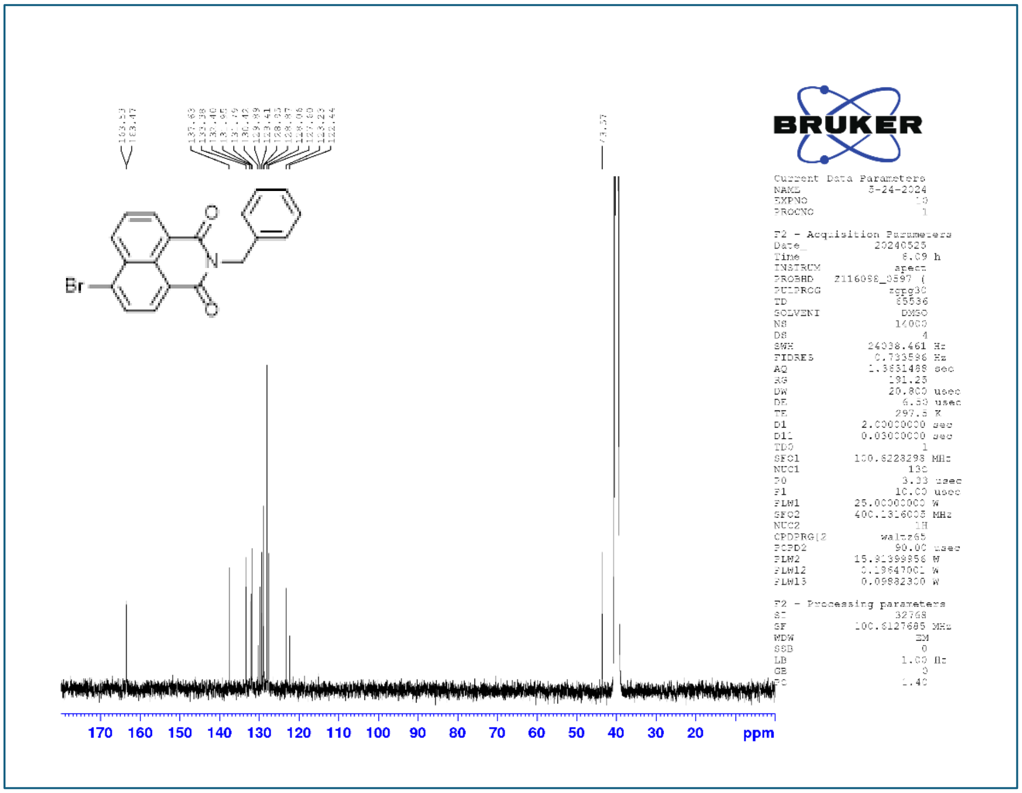
Br
O
N
BRUKER
Current Data Parameters
5-24-2024
F2 - Acquisition Parameters
F2 - Processing parameters
170 160 150 140 130 120 110 100 90 80 70 60 50 40 30 20 ppm

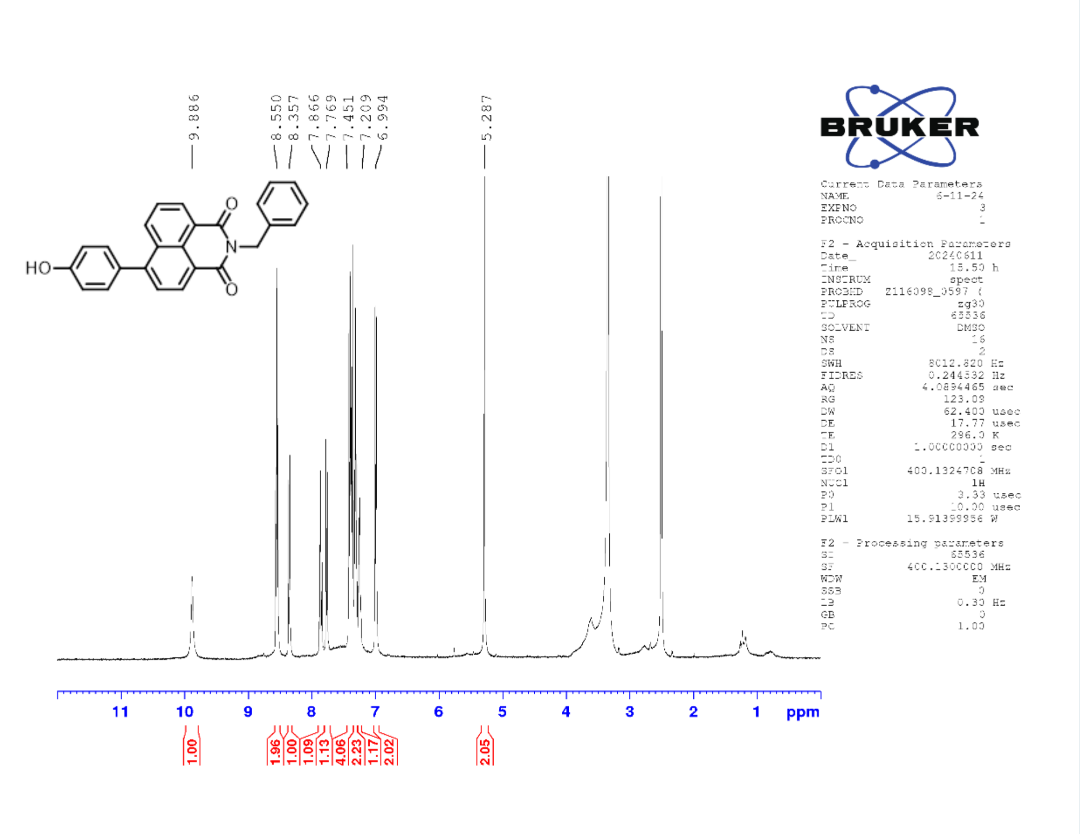
9.886
8.550
8.357
7.866
7.769
7.451
7.209
6.994
5.287
HO
N
O
O
BRUKER
Current Data Parameters
NAME 6-11-24
EXPNO 3
PROCNO 1
F2 - Acquisition Parameters
Date_ 20240611
Time 15.50 h
INSTRUM spect
PROBHD Z116098_0597 (
PULPROG zg30
TD 65536
SOLVENT DMSO
NS 16
DS 2
SWH 8012.820 Hz
FIDRES 0.244532 Hz
AQ 4.0894465 sec
RG 123.09
DW 62.400 usec
DE 17.77 usec
TE 296.0 K
D1 1.00000000 sec
TD0 1
SFO1 400.1324708 MHz
NUC1 1H
P0 3.33 usec
P1 10.00 usec
PLW1 15.91399956 W
F2 - Processing parameters
SI 65536
SF 400.1300000 MHz
WDW EM
SSB 0
LB 0.30 Hz
GB 0
PC 1.00
11 10 9 8 7 6 5 4 3 2 1 ppm
1.00
1.96
1.00
1.09
1.13
4.06
2.23
1.17
2.02
2.05

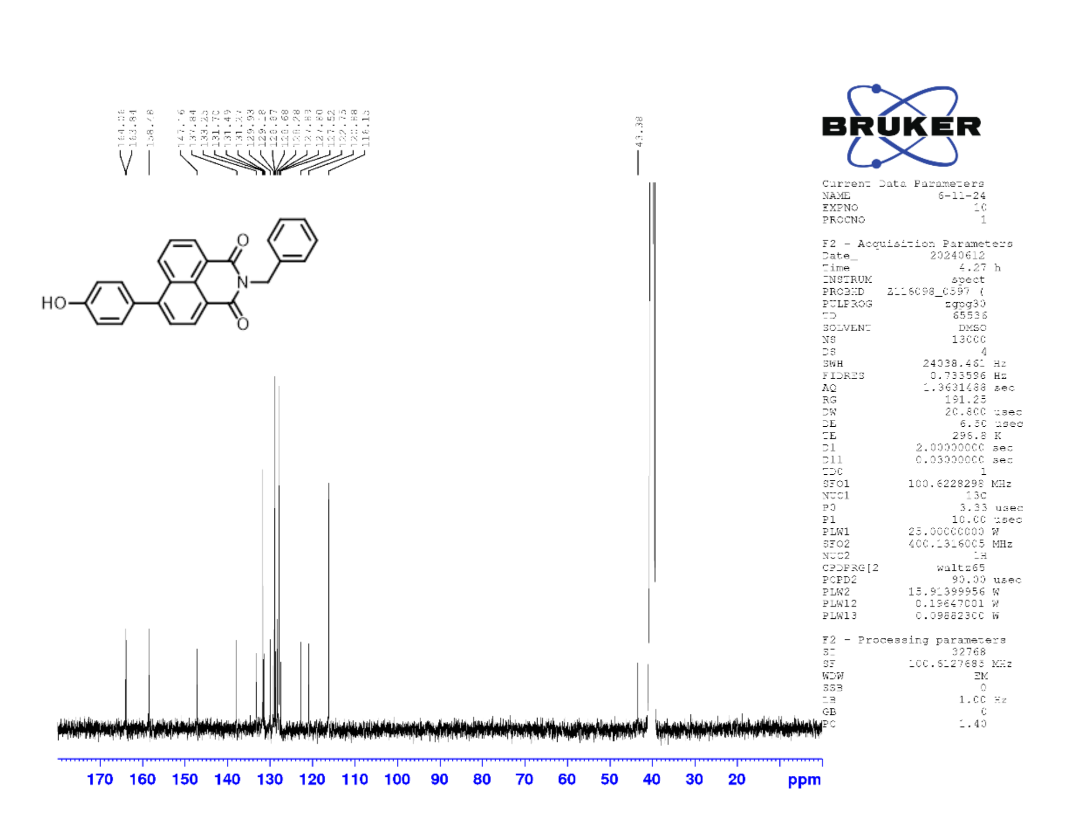
43.38
HO
N
O
O
BRUKER
Current Data Parameters
NAME 6-11-24
EXPNO 10
PROCNO 1
F2 - Acquisition Parameters
Date_ 20240612
Time 4.27 h
INSTRUM spect
PROBHD Z116098_0597 (
PULPROG zgpg30
TD 65536
SOLVENT DMSO
NS 13000
DS 4
SWH 24038.461 Hz
FIDRES 0.733596 Hz
AQ 1.3631488 sec
RG 191.25
DW 20.800 usec
DE 6.50 usec
TE 296.8 K
D1 2.00000000 sec
D11 0.03000000 sec
TD0 1
SFO1 100.6228298 MHz
NUC1 13C
P0 3.33 usec
P1 10.00 usec
PLW1 25.00000000 W
SFO2 400.1316005 MHz
NUC2 1H
CPDPRG[2 waltz65
PCPD2 90.00 usec
PLW2 15.91399956 W
PLW12 0.19647001 W
PLW13 0.09882300 W
F2 - Processing parameters
SI 32768
SF 100.6127685 MHz
WDW EM
SSB 0
LB 1.00 Hz
GB 0
PC 1.40
170 160 150 140 130 120 110 100 90 80 70 60 50 40 30 20 ppm

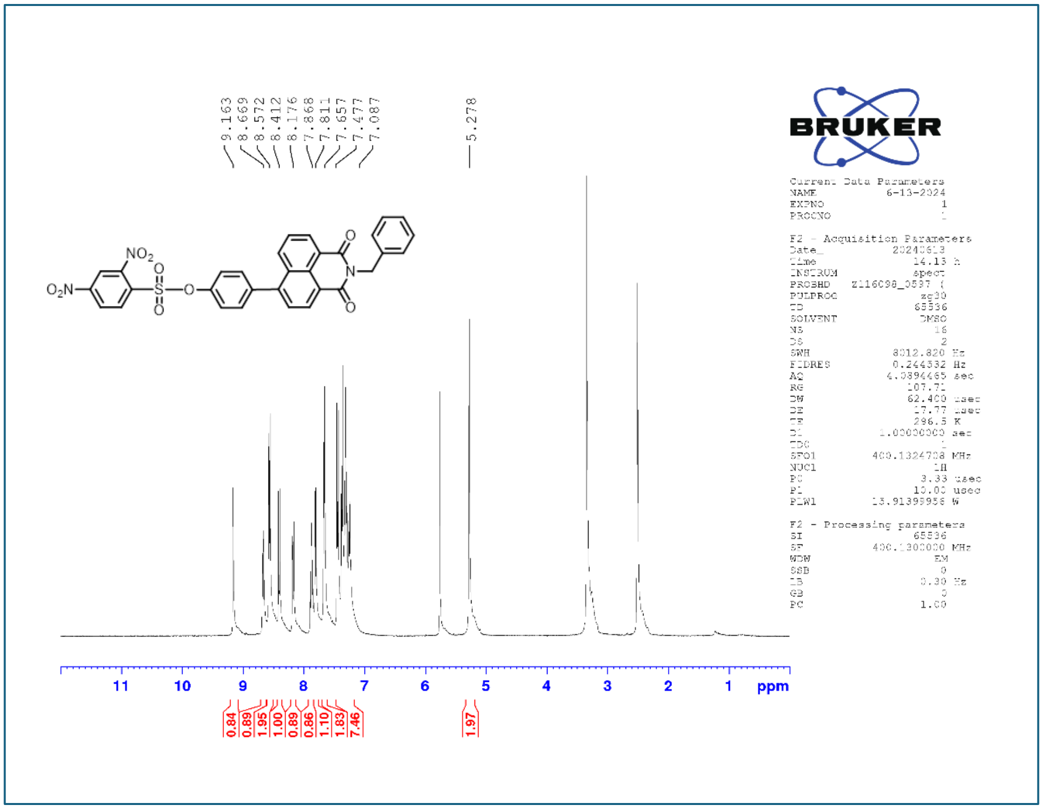
9.163
8.669
8.572
8.412
8.176
7.868
7.811
7.657
7.477
7.087
5.278
BRUKER
11 10 9 8 7 6 5 4 3 2 1 ppm
0.84 0.89 1.95 1.00 0.89 0.86 1.10 1.83 7.46
1.97

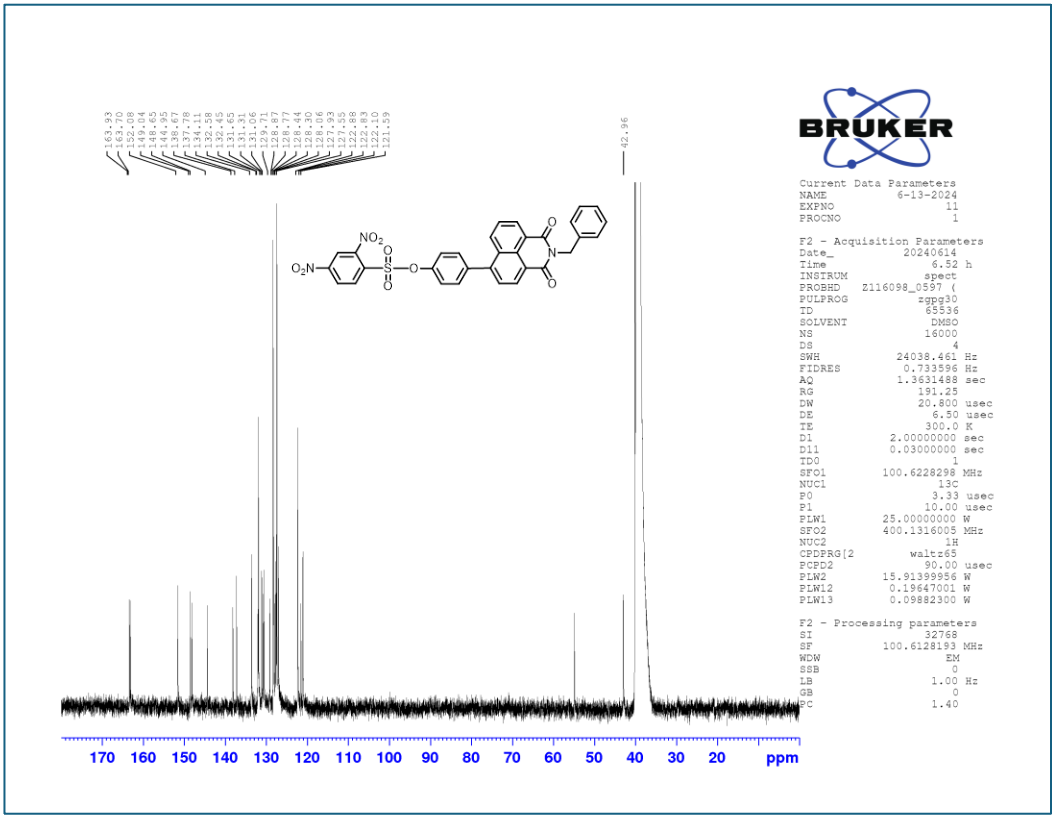
163.93
163.70
152.08
149.04
148.65
144.95
138.67
137.78
134.11
132.58
132.45
131.65
131.31
131.06
129.71
128.87
128.77
128.44
128.30
128.06
127.93
127.55
122.88
122.83
122.10
121.59
42.96
BRUKER
Current Data Parameters
NAME 6-13-2024
EXPNO 11
PROCNO 1
F2 - Acquisition Parameters
Date_ 20240614
Time 6.52 h
INSTRUM spect
PROBHD Z116098_0597 (
PULPROG zgpg30
TD 65536
SOLVENT DMSO
NS 16000
DS 4
SWH 24038.461 Hz
FIDRES 0.733596 Hz
AQ 1.3631488 sec
RG 191.25
DW 20.800 usec
DE 6.50 usec
TE 300.0 K
D1 2.00000000 sec
D11 0.03000000 sec
TD0 1
SFO1 100.6228298 MHz
NUC1 13C
P0 3.33 usec
P1 10.00 usec
PLW1 25.00000000 W
SFO2 400.1316005 MHz
NUC2 1H
CPDPRG[2 waltz65
PCPD2 90.00 usec
PLW2 15.91399956 W
PLW12 0.19647001 W
PLW13 0.09882300 W
F2 - Processing parameters
SI 32768
SF 100.6128193 MHz
WDW EM
SSB 0
LB 1.00 Hz
GB 0
PC 1.40
170 160 150 140 130 120 110 100 90 80 70 60 50 40 30 20 ppm

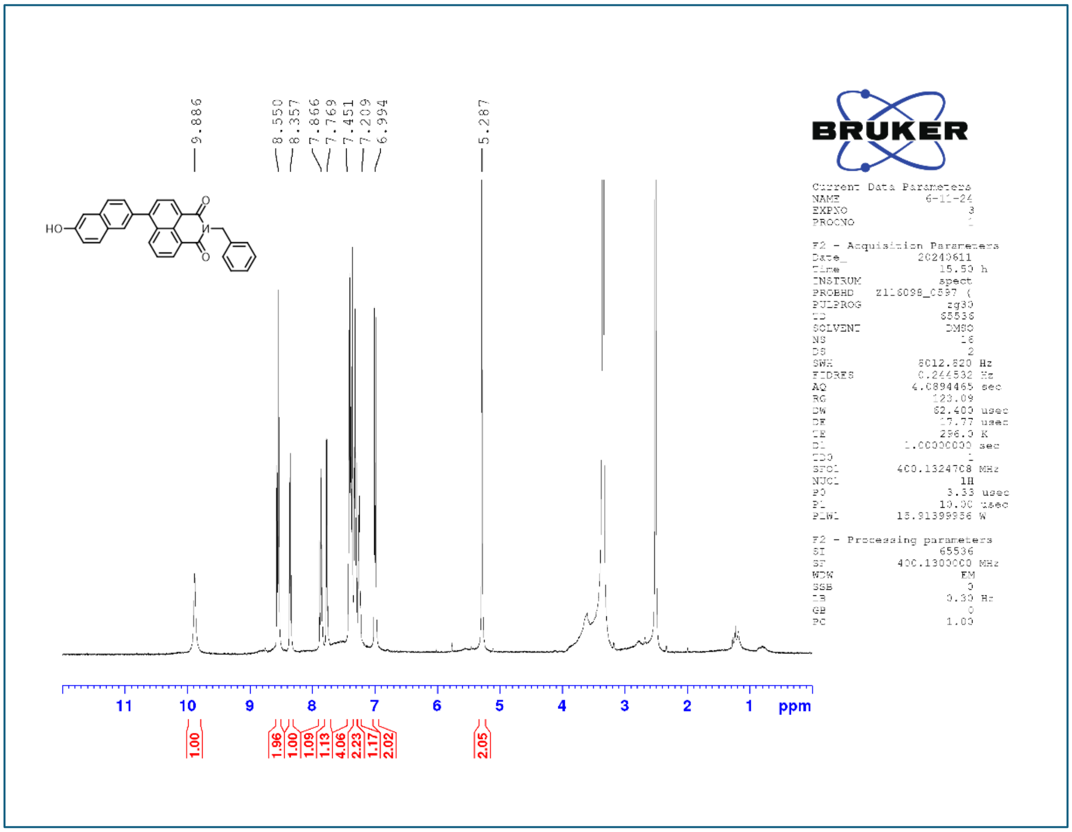
9.886
8.550
8.357
7.866
7.769
7.451
7.209
6.994
5.287
HO
N
O
O
BRUKER
Current Data Parameters
NAME 6-11-24
EXPNO 3
PROCNO 1
F2 - Acquisition Parameters
Date_ 20240611
INSTRUM spect
PULPROG zg30
TD 65536
SOLVENT DMSO
NS 16
DS 2
SFO1 400.1324708 MHz
NUC1 1H
F2 - Processing parameters
SI 65536
SF 400.1300000 MHz
WDW EM
11 10 9 8 7 6 5 4 3 2 1 ppm
1.00
1.96
1.00
1.09
1.13
4.06
2.23
1.17
2.02
2.05

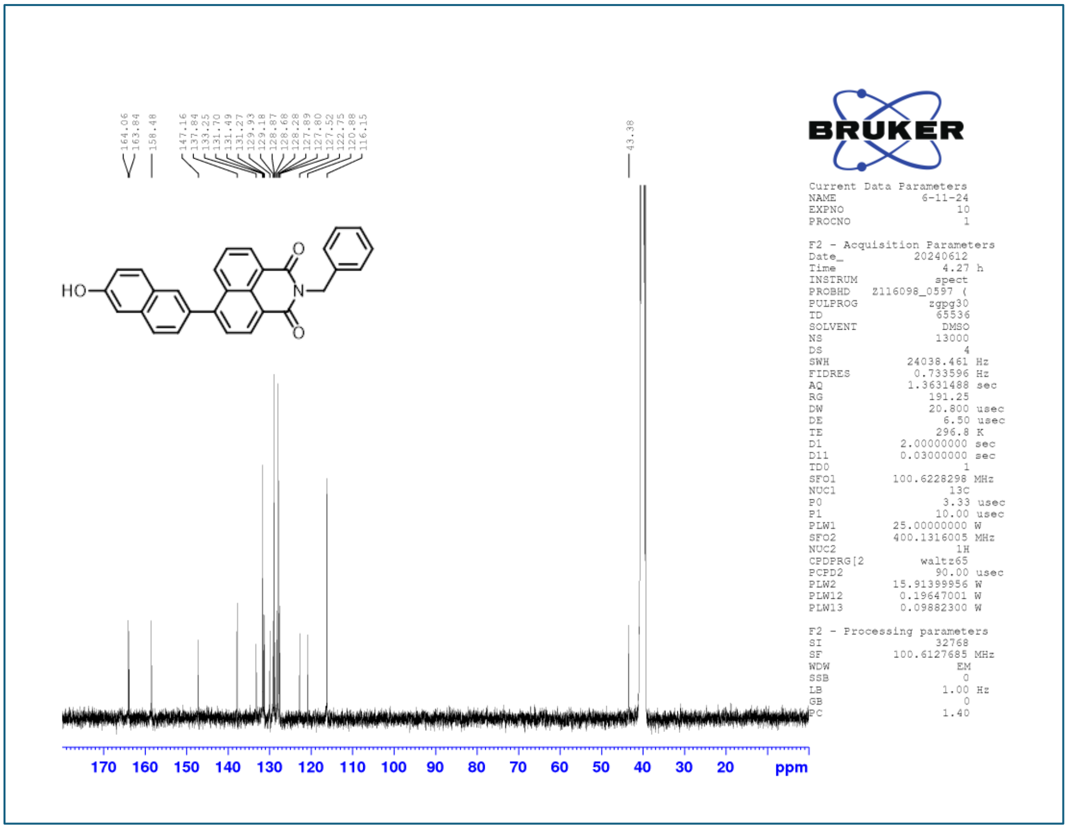
164.06
163.84
158.48
147.16
137.84
133.25
131.70
131.49
131.27
129.93
129.18
128.87
128.68
128.28
127.89
127.80
127.52
122.75
120.88
116.15
43.38
HO
N
O
O
BRUKER
Current Data Parameters
NAME 6-11-24
EXPNO 10
PROCNO 1
F2 - Acquisition Parameters
Date_ 20240612
Time 4.27 h
INSTRUM spect
PROBHD Z116098_0597 (
PULPROG zgpg30
TD 65536
SOLVENT DMSO
NS 13000
DS 4
SWH 24038.461 Hz
FIDRES 0.733596 Hz
AQ 1.3631488 sec
RG 191.25
DW 20.800 usec
DE 6.50 usec
TE 296.8 K
D1 2.00000000 sec
D11 0.03000000 sec
TD0 1
SFO1 100.6228298 MHz
NUC1 13C
P0 3.33 usec
P1 10.00 usec
PLW1 25.00000000 W
SFO2 400.1316005 MHz
NUC2 1H
CPDPRG[2 waltz65
PCPD2 90.00 usec
PLW2 15.91399956 W
PLW12 0.19647001 W
PLW13 0.09882300 W
F2 - Processing parameters
SI 32768
SF 100.6127685 MHz
WDW EM
SSB 0
LB 1.00 Hz
GB 0
PC 1.40
170 160 150 140 130 120 110 100 90 80 70 60 50 40 30 20 ppm

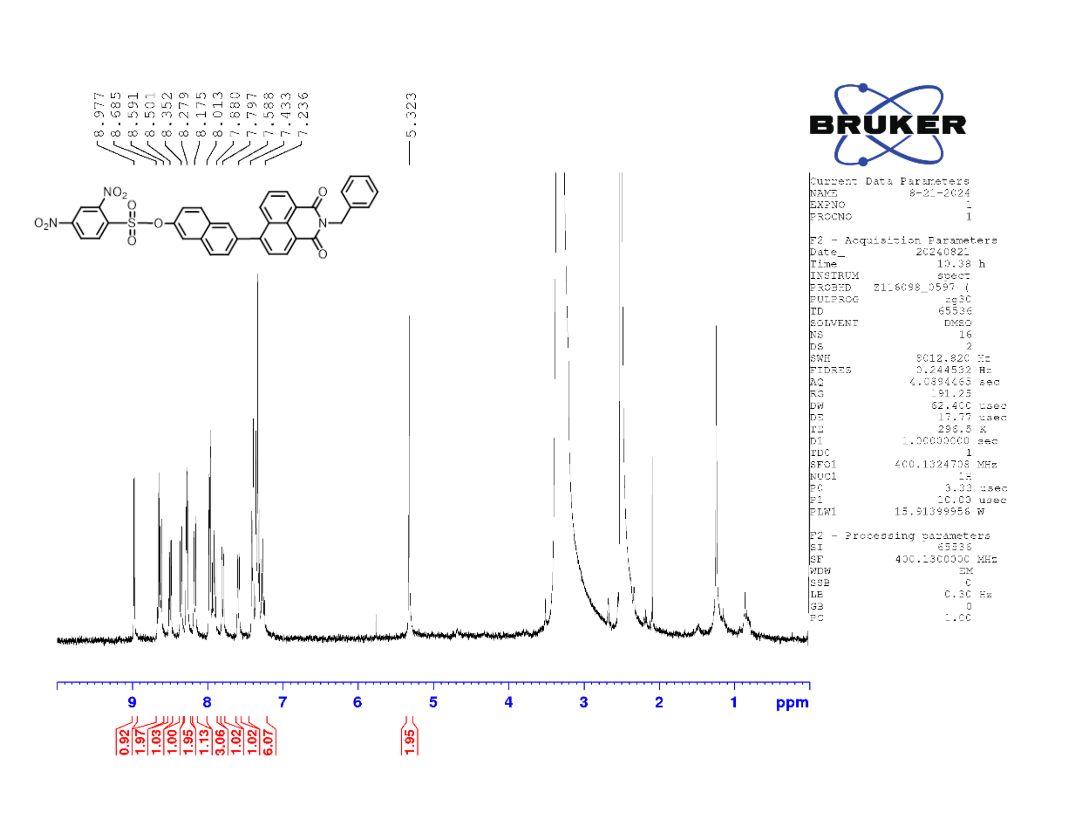

8.977
8.685
8.591
8.501
8.352
8.279
8.175
8.013
7.880
7.797
7.588
7.433
7.236
5.323
NO_2
O_2N
S
O
N
BRUKER
Current Data Parameters
NAME 8-21-2024
EXPNO 1
PROCNO 1
F2 - Acquisition Parameters
Date_ 20240821
Time 10.38 h
INSTRUM spect
PULPROG zg30
TD 65536
SOLVENT DMSO
NS 16
DS 2
SWH 8012.820 Hz
FIDRES 0.244532 Hz
AQ 4.0894465 sec
RG 191.25
DW 62.400 usec
DE 17.77 usec
TE 298.5 K
D1 1.00000000 sec
TD0 1
SFO1 400.1324708 MHz
NUC1 1H
P0 3.33 usec
P1 10.00 usec
PLW1 15.91399956 W
F2 - Processing parameters
SI 65536
SF 400.1300000 MHz
WDW EM
SSB 0
LB 0.30 Hz
GB 0
PC 1.00
9
8
7
6
5
4
3
2
1
ppm
0.92
1.97
1.03
1.00
1.95
1.13
3.06
1.02
1.02
6.07
1.95

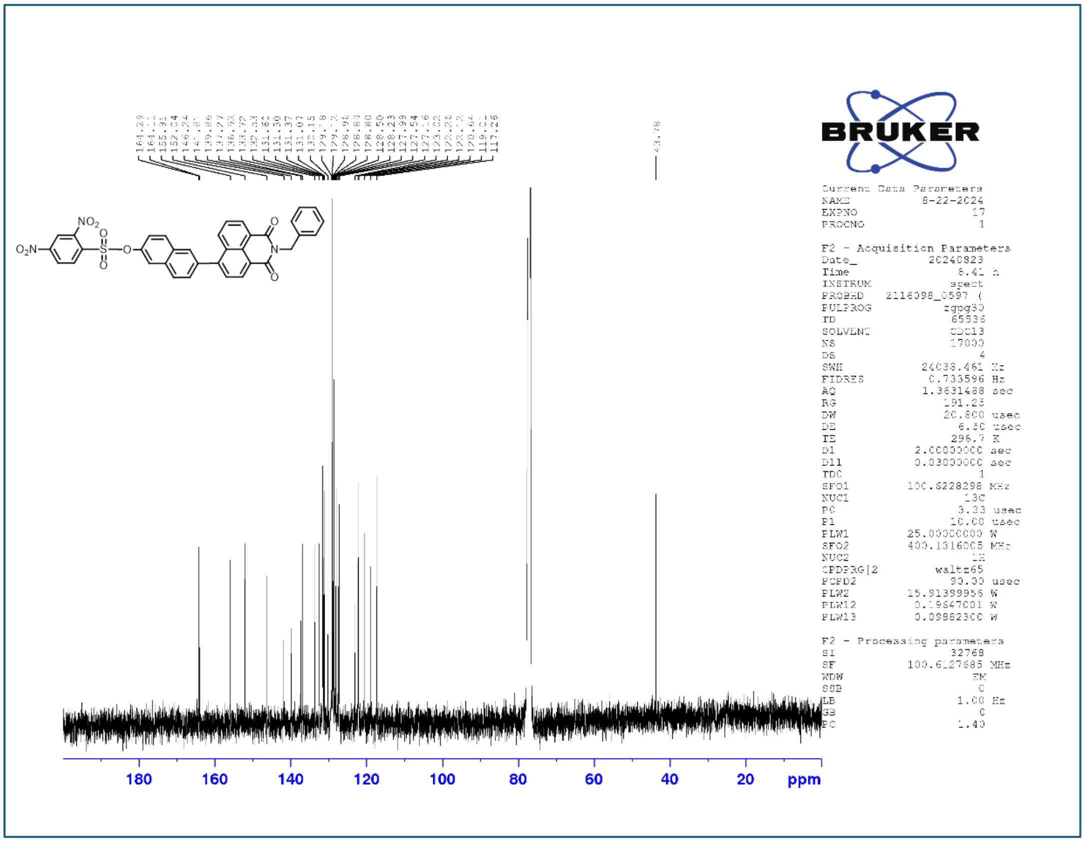

164.29
155.91
152.04
146.24
139.86
137.27
133.72
132.53
131.50
131.37
131.07
128.96
128.80
128.50
128.23
127.99
127.54
123.02
120.64
117.26
43.78
NO_2
O_2N
S
O
N
BRUKER
Current Data Parameters
NAME 8-22-2024
EXPNO 17
PROCNO 1
F2 - Acquisition Parameters
Date_ 20240823
Time 6.41 h
INSTRUM spect
PULPROG zgpg30
TD 65536
SOLVENT CDCl3
NS 17000
DS 4
SWH 24038.461 Hz
FIDRES 0.733596 Hz
AQ 1.3631488 sec
RG 191.25
DW 20.800 usec
DE 6.50 usec
TE 296.7 K
D1 2.00000000 sec
D11 0.03000000 sec
TD0 1
SFO1 100.6228298 MHz
NUC1 13C
P0 3.33 usec
P1 10.00 usec
PLW1 25.00000000 W
SFO2 400.1316005 MHz
NUC2 1H
CPDPRG[2 waltz65
PCPD2 90.00 usec
PLW2 15.91399956 W
PLW12 0.19647001 W
PLW13 0.09882300 W
F2 - Processing parameters
SI 32768
SF 100.6127685 MHz
WDW EM
SSB 0
LB 1.00 Hz
GB 0
PC 1.40
180
160
140
120
100
80
60
40
20
ppm

Table S1: The comparison of detection parameters of **M1** with other H_2S probes.

	**Detection Limit**	**Detection linear range**	**Reponse time**	**Ref**
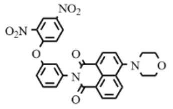	9.8 nM	0-12 μM	15 minutes	(1)
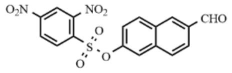	0.851 nM	0-20 μM	11 minutes	(2)
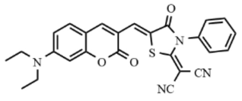	0.98 μM	0-44 μM	------	(3)
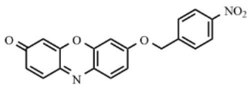	17.3 μM	0-5 mM	4 hours	(4)
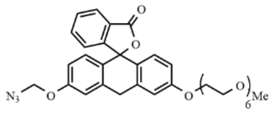	3.99 μM	0-0.8 mM	20 minutes	(5)
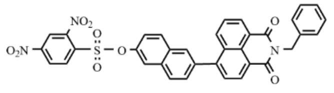	0.64 μM	0-228 μM	10 minutes	**This work**

Reference:

38 Wan, D.; Pan, T.; Ou, P.; Zhou, R.; Ouyang, Z.; Luo, L.; Xiao, Z.; Peng, Y. Construct a lysosome-targeting and highly selective fluorescent probe for imaging of hydrogen sulfide in living cells and inflamed tissues. *Spectrochimica Acta Part A: Molecular and Biomolecular Spectroscopy*, **2021**, 249,119311.

39. Wang, H.; Li, Y.; Yang, S.; Tian, H.; Liang, S.; Sun, B. Dual-function fluorescent probe for detection of hydrogen sulfide and water content in dimethyl sulfoxide. *ACS Omega* **2019**, 4, 10695−10701.

40. Shang,Z.; Meng, Q.; Tian,D.; Wang,Y.; Zhang, Z.; Zhang, Z.; Zhang, R. Red-emitting fluorescent probe for hydrogen sulfide detection and its applications in food freshness determination and in vivo bioimaging. *Food Chemistry*, **2023**,427, 136701.

41. Sun, Y.; Tang, X.; Zhang, K.; Liu, K.; Li, Z.; Zhao,L. Hydrogen sulfide detection and zebrafish imaging by a designed sensitive and selective fluorescent probe based on resorufin, *Spectrochimica Acta Part A: Molecular and Biomolecular Spectroscopy*, **2022**, 120265.

42. Feng, W.; Xiao, Q.; Wang, L.; Yang, Y. A new fluorescent probe for hydrogen sulfide detection in solution and living cells. *Molecules* **2023**, 28, 6195.
